# Electrocardiographic Abnormalities in Nigerian Hypertensives With Echocardiographic Left Ventricular Hypertrophy

**DOI:** 10.7759/cureus.60170

**Published:** 2024-05-12

**Authors:** Olugbenga O Abiodun, Tina Anya, Victor T Adekanmbi

**Affiliations:** 1 Internal Medicine/Cardiology, Federal Medical Centre, Abuja, NGA; 2 Obstetrics and Gynecology, University of Texas Medical Branch at Galveston, Galveston, USA

**Keywords:** nigerian, essential hypertension, left ventricular hypertrophy, echocardiographic, electrocardiographic

## Abstract

Introduction

To enhance the diagnosis of anatomic left ventricular hypertrophy (LVH) using electrocardiography (ECG), we aimed to identify common ECG amplitude and non-amplitude abnormalities in Nigerian patients with hypertensive echocardiographic LVH.

Method

The study included 1,765 patients with essential hypertension aged 18 years and older from the Federal Medical Centre Abuja Hypertension Registry (FMCAHR). Participants underwent echocardiography and ECG following the American College of Cardiology and the American Society of Echocardiography guidelines.

Results

The prevalence of overall ECG LVH amplitude criteria (43.8%) and individual criteria of Cornell voltage (27.1%), Sokolow-Lyon voltage (23.2%), and Gubner-Ungerleider (13.9%) were higher than non-amplitude ECG abnormalities among patients with echocardiographic LVH. The sensitivity and specificity of LVH criteria were 43.8% and 79.5% for overall ECG LVH, 23.2% and 87.2% for Sokolow-Lyon voltage, 27.1% and 93.3% for Cornell voltage, and 13.9% and 95.4% for Gubner-Ungerleider criteria, respectively.

After multivariable adjustment, non-amplitude ECG changes, including prolonged corrected QT (QTc) (odds ratio (OR): 1.68, 95% confidence interval (CI): 1.06-2.66), left ventricular (LV) strain pattern (OR: 1.83, CI: 1.23-2.72), left axis deviation (OR: 1.56, CI: 1.09-2.24), poor R wave progression (OR: 2.36, CI: 1.40-3.97), premature ventricular contractions (OR: 1.80, CI: 1.10-2.91), premature atrial contractions (OR: 2.06, CI: 1.10-3.87), atrial fibrillation (OR: 2.40, CI: 1.20-4.82), and left atrial abnormality (OR: 8.43, CI: 2.95-24.05), were associated with echocardiographic LVH (p < 0.05).

Conclusion

In our cohort of hypertensive patients, ECG LVH amplitude criteria were the most frequently observed abnormalities associated with echocardiographic LVH. Our findings suggest that despite the low sensitivity, ECG LVH amplitude criteria may remain valuable in diagnosing echocardiographic LVH.

## Introduction

Left ventricular hypertrophy (LVH) is a significant independent predictor of cardiovascular (CV) mortality and morbidity in both hypertensive individuals and the general population [1,2]. It increases the risk of heart failure (HF), coronary heart disease (CHD), arrhythmias, and sudden cardiac death (SCD) [1-4]. This risk applies not only to echocardiographic LVH but also to ECG LVH, which provides independent prognostic information [1]. ECG LVH is linked with CV events, CV mortality, and all-cause mortality [5]. Given that an easily accessible and inexpensive screening tool like ECG accurately provides prognostic information on LVH, there continues to be considerable interest in researching the ECG to aid the diagnosis of LVH. The poor sensitivity of ECG amplitude criteria is well documented, and it has been suggested that relying on a single ECG parameter to identify LVH may be impractical [6]. Conditions other than hypertension cause an increase in left ventricular mass (LVM) leading to heterogenous pathological features, such as cardiomyocyte hypertrophy, interstitial fat infiltration, and extracellular amyloid deposition, among others [6]. Additionally, genetic factors, epigenetic modifications, and various adaptive responses may contribute to heterogeneous ECG amplitude responses in different individuals with the same LVH etiology [6]. Therefore, the future of the ECG in the diagnosis and prognostication of LVH has been suggested to lay in the complimentary use of the amplitude and non-amplitude ECG changes that result from the increase in LVM [6].

To identify common non-amplitude and amplitude ECG abnormalities, we analyzed data from the Federal Medical Centre Abuja Hypertension Registry (FMCAHR). These findings have the potential to be developed into diagnostic scores to enhance the diagnostic accuracy of ECG for echocardiographic LVH in hypertensive patients. Furthermore, the integration of these abnormalities could aid in assessing CV risks, thereby contributing to the reduction of CV events.

## Materials and methods

Participants

**Figure 1:**
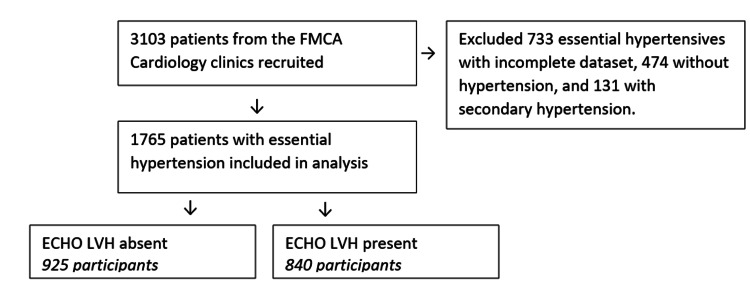
Flow data profile. ECHO LVH: echocardiographic left ventricular hypertrophy; FMCA: Federal Medical Centre, Abuja.

The FMCAHR is a prospective registry that consecutively enrolled patients attending the cardiology clinics of Federal Medical Centre, Abuja (FMCA) from 2016 to 2021. The FMCA is a tertiary healthcare center in the heart of the capital city of Nigeria, Abuja. Initially, physicians collected data on biodata, anthropometry, past medical history, family and social history, drug history, comorbidities, blood pressure (BP) measurements, blood and urine tests, ECG, echocardiography, and medications. During follow-up visits, updates on BP, blood and urine tests, medications, new comorbidities, and CV complications were recorded. After excluding those with secondary hypertension, non-hypertensives, and those with incomplete datasets, 1,765 patients with essential hypertension, aged 18 years and above were included in this study (Figure 1).

All patients provided informed consent and the study received ethics clearance with approval number FMCABJ/HREC/2017/009 from the hospital’s ethics research committee.

Electrocardiography

Resting 12-lead ECG was performed on all patients during their first visit by trained technicians according to the American College of Cardiology guidelines [7]. The Schiller AT-102 system with serial number 070.07911 (Baar, Switzerland), set at a speed of 25 mm/s and 10 mm/mV calibration, was used, and ECGs were manually reported by the first and second authors, who are experienced cardiologists.

Echocardiography

Echocardiography was performed and reported by the same cardiologists during the patients’ visits using either the General Electric Vivid E9, GA314809-03 (Boston, MA) or General Electric, Model S6, 050140VS6N. Measurements were taken per recommendations of the American Society of Echocardiography [8]. Left ventricular internal diameter at diastole (LVIDD), left ventricular posterior wall thickness at end-diastole (PWTD), and interventricular septal thickness at end-diastole (IVSTD) were measured to calculate left ventricular mass index (LVMI) [8].

Definitions

LVH is defined as LVMI > 95 g/m^2^ in females and >115 g/m^2^ in males, according to the American Society of Echocardiography [8]. LVMI was calculated using the linear cube formula of the American College of Cardiology: 0.8 (1.04 ([LVIDD + PWTD + IVSTD]^3^- [LVIDD]^3^))+ 0.6 g and indexing with body surface area (Dubois method) [1,8]. Relative wall thickness (RWT) was calculated with the formula: 2 × PWTD/LVIDD [1].

CV comorbidities were diagnosed by hospital physicians using appropriate tests, following guideline recommendations, as the presence of HF, chronic kidney disease (CKD), cerebrovascular disease (CVD), coronary artery disease (CAD), and arrhythmias at recruitment or during follow-up.

Abnormal Q waves are defined as Q waves >0.04 sec, >2 mm, >25% of the depth of QRS complex, and seen in leads V1-3.

Atrial fibrillation (AFib) is defined as absent P waves, fibrillary waves, and irregularly irregular R-R interval, with an atrial rate of 300-600 bpm.

ECG LVH is defined as the presence of any of the following criteria: Sokolow-Lyon voltage (SKL) - SV1 + RV5 or RV6 >35 mm, Cornell voltage - SV3 + RaVL > 20mm (female), SV3 + RaVL > 28mm (male), or Gubner-Ungerleider (GU) - RI + SIII > 25 mm.

First-degree atrioventricular (AV) block (1°AVB) is PR interval > 200 ms. Left atrial abnormality (LAA) is a bifid P wave with peak-to-peak interval >0.04 sec and P wave duration >0.12 sec in lead II, and p terminal force of ≥0.04 mm in lead V1. Left axis deviation (LAD) is defined as a QRS axis of -30° to -90° using the hexaxial reference system. The left bundle branch block (LBBB) is defined as QRS > 155 ms [9].

Non-specific intra-ventricular conduction defect (NICD) is QRS > 120 ms in the absence of right bundle branch block (RBBB) or LBBB morphology. Poor R wave progression (PRWP) is R wave ≤ 3 mm in lead V3.

Premature atrial contraction (PAC) is defined as a premature abnormal P wave with QRS < 120 ms. Premature ventricular contractions (PVC) are defined as premature beat, absent or retrograde P wave, QRS > 120 ms, with discordant ST-T wave, and a full compensatory pause.

Prolonged QTc interval is >0.45 seconds for males and ≥0.46 seconds for females [10]. QT was measured using leads II or V5 from the start of the QRS complex to the end of the T wave determined by the tangent technique [11], and QT was corrected using the Hodges formula [12]. 

RBBB is defined as incomplete (QRS duration >110 ms) or complete (QRS duration >120 ms), with RSR’ pattern in V1-V3.

Short PR interval is PR interval < 120 ms, with QRS < 120 ms. Sinus tachycardia is sinus rhythm with a heart rate > 100 beats per minute (bpm). Sinus bradycardia is sinus rhythm with a heart rate < 60 bpm.

LV strain pattern is defined as ST depression and T wave inversion in I, aVL, and V5-V6. ST-T changes are defined as straightening of the ST segment, ST-segment depression or elevation, flattening of the T wave, biphasic T waves, or T-wave inversion.

Data analysis

Categorical variables are expressed as proportions and percentages, while continuous variables are expressed as means ± standard deviation. The associations between patient characteristics, ECG abnormalities, and echocardiographic LVH were tested by chi-square and independent t-test. Fisher’s exact test was applied for expected cell sizes of less than 10. Sensitivity and specificity testing were done using chi-square and the receiver operating characteristics (ROC) curve. Multivariable logistic regression with model-fitting statistics was employed to establish associations between three ECG LVH criteria and echocardiographic LVH. Data were analyzed using the SPSS version 26 software for Windows (IBM Corp., Armonk, NY). P-value < 0.05 was taken as statistically significant.

## Results

Table 1 presents the baseline characteristics of participants by the presence or absence of echocardiographic LVH. The mean age of the participants was 55 ± 13 years, with 983 females (55.7%) and 782 males (44.3%). Participants with echocardiographic LVH were significantly older at 57.0 ± 12.8 years (p < 0.001). More females had echocardiographic LVH than males (p = 0.007) and there were 186 (22.1%) diabetes mellitus patients with LVH compared to 169 (18.3%) without (p = 0.04). Office systolic blood pressure (p = 0.001) and diastolic blood pressure (p = 0.005) were significantly higher in patients with LVH compared to those without and there was a significantly higher LVMI of 136.9 ± 36.0 g/m^2^ in the LVH group (p < 0.001). Estimated glomerular filtration rate (eGFR) was significantly lower in the LVH group (p = 0.001) while CV comorbidities were found in 284 (33.8%) of the LVH group compared to 91 (9.8%) without LVH (p < 0.001). No significant differences were observed in BMI, duration of hypertension, alcohol intake, tobacco use, fasting blood glucose (FBG), cholesterol levels, and the number of hypertensive medications between the two groups (p > 0.05).

**Table 1:** Baseline characteristics of participants by echocardiographic LVH. BMI: body mass index; DBP: diastolic blood pressure; ECHO LVH: echocardiographic left ventricular hypertrophy; eGFR: estimated glomerular filtration rate; HDL: high-density lipoprotein; LDL: low-density lipoprotein; LVH: left ventricular hypertrophy; SBP: systolic blood pressure.

Variables	Total (n = 1765)	ECHO LVH absent (n = 925)	ECHO LVH present (n = 840)	P-value
Age (years)	55 ± 13	53.6 ± 13.1	57.0 ± 12.8	<0.001
Sex				
Male	782 (44.3%)	438 (47.4%)	344 (29.1%)	0.007
Female	983 (55.7%)	487 (52.7%)	496 (59.1%)	
BMI (kg/m^2^)	30.4 ± 6.0	30.4 ± 5.7	30.4 ± 6.2	0.09
Duration of hypertension (years)	8.4 ± 8.4	8.2 ± 8.4	8.6 ± 8.4	0.96
Diabetes mellitus	355 (20.2%)	169 (18.3%)	186 (22.1%)	0.04
Alcohol use	554 (31.4%)	303 (32.8%)	251 (29.9%)	0.42
Tobacco use	109 (6.2%)	59 (6.4%)	50 (6.0%)	0.88
First visit office SBP (mmHg)	147 ± 22	145.8 ± 20.4	148.1 ± 24.4	0.001
First visit office DBP (mmHg)	90 ± 26	89 ± 12.6	90 ± 34.9	0.005
Relative wall thickness	0.49 ± 0.1	0.50 ± 0.1	0.49 ± 0.1	0.002
Left ventricular mass index (g/m^2^)	109.5 ± 37.8	84.7 ± 16.0	136.9 ± 36.0	<0.001
Fasting blood glucose (mmol/l)	6.0 ± 2.4	5.9 ± 2.2	6.2 ± 2.6	0.65
eGFR (ml/min/1.73m^2^)	72.6 ± 20.0	74.1 ± 18.8	70.9 ± 21.1	0.001
Total cholesterol (mmol/l)	5.1 ± 1.26	5.2 ± 1.2	5.0 ± 1.3	0.58
HDL cholesterol (mmol/l)	1.6 ± 0.6	1.6 ± 0.6	1.6 ± 0.6	0.08
LDL cholesterol (mmol/l)	3.1 ± 1.1	3.1 ± 1.1	3.0 ± 1.1	0.99
Triglycerides (mmol/l)	1.2 ± 0.6	1.2 ± 0.6	1.2 ± 0.6	0.83
Number of antihypertensives	2.7 ± 1.2	2.4 ± 1.1	2.9 ± 1.1	0.34
Cardiovascular comorbidities	375 (21.3%)	91 (9.8%)	284 (33.8%)	<0.001

Table 2 compares ECG abnormalities between participants with and without echocardiographic LVH. The most common abnormality in those with echocardiographic LVH was ECG LVH in 43.8% of participants (p < 0.001). Additionally, 27.1% with Cornell voltage criterion, 23.2% with SKL, and 13.9% with GU criterion had echocardiographic LVH (p < 0.001). These were followed by 11.8% with LAD, 9.2% with LV strain pattern, 8.3% with PRWP, 7.4% with prolonged QTc, and 7.3% with premature ventricular contractions (PVCs) (p < 0.001). Other abnormalities included 4.8% with LAA (p < 0.001), 3.7% with PACs (p = 0.01), 3.6% with AFib (p = 0.002), 2.0% with abnormal Q waves (p = 0.02), and 0.8% with LBBB (p = 0.031). First-degree AVB, sinus tachycardia, ST-T changes, sinus bradycardia, RBBB, NICD, and short PR interval were equally seen in both groups (p > 0.05).

**Table 2:** Electrocardiographic abnormalities in echocardiographic LVH. AFib: atrial fibrillation; AVB: atrioventricular block; ECG LVH: electrocardiographic left ventricular hypertrophy; ECHO LVH: echocardiographic left ventricular hypertrophy; NICD: nonspecific intraventricular conduction defect; LAA: left atrial abnormality; LAD: left axis deviation; LBBB: left bundle branch block; PACs: premature atrial contractions; PRWP: poor R wave progression; PVCs: premature ventricular contractions; RBBB: right bundle branch block. * Fisher’s exact test.

Variables	ECHO LVH absent	ECHO LVH present	P-value
	n = 925	n = 840	
ECG LVH	190 (20.5%)	368 (43.8%)	<0.001
Cornell voltage	62 (6.7%)	228 (27.1%)	
Sokolow-Lyon	118 (12.8%)	195 (23.2%)	
Gubner-Ungerleider	43 (4.6%)	117 (13.9%)	
LAD	57 (6.2%)	99 (11.8%)	<0.001
Strain pattern	44 (4.8%)	77 (9.2%)	<0.001
First-degree AVB	61 (6.6%)	72 (8.6%)	0.12
Sinus tachycardia	77 (8.3%)	71 (8.5%)	0.92
PRWP	22 (2.4%)	70 (8.3%)	<0.001
ST-T changes	58 (6.3%)	64 (7.6%)	0.27
Prolonged QTc	32 (3.5%)	62 (7.4%)	<0.001
PVCs	28 (3.0%)	61 (7.3%)	<0.001
Sinus bradycardia	73 (7.9%)	53 (6.3%)	0.20
LAA	4 (0.4%)	40 (4.8%)	<0.001
PACs	16 (1.7%)	31 (3.7%)	0.01
AFib	12 (1.3%)	30 (3.6%)	0.002
RBBB	25 (2.7%)	30 (3.6%)	0.29
Abnormal Q	7 (0.8%)	17 (2.0%)	0.02
NICD	9 (1.0%)	15 (1.8%)	0.14
Short PR interval	9 (1.0%)	13 (1.5%)	0.28
LBBB	1 (0.1%)	7 (0.8%)	*0.031

Table 3 shows the sensitivities and specificities of ECG criteria for diagnosing echocardiographic LVH. The ECG’s overall sensitivity for detecting echocardiographic LVH was 43.8%, with a specificity of 79.5% (p < 0.001). Sensitivities were 13.9% for GU, 23.2% for SKL voltage, and 27.1% for Cornell voltage criteria (p < 0.001). Specificities for SKL voltage, Cornell voltage, and GU were 87.2%, 93.3%, and 95.4%, respectively (p < 0.001). All ECG criteria, collectively and individually, showed an area under the curve of <0.63.

**Table 3:** Sensitivity and specificity of ECG criteria. AOC: area under the curve; ECG LVH: combined ECG left ventricular hypertrophy criteria.

Variable	Sensitivity	Specificity	P-value	AOC
ECG LVH	43.8%	79.5%	<0.001	0.62
Sokolow-Lyon criterion	23.2%	87.2%	<0.001	0.55
Cornell voltage criterion	27.1%	93.3%	<0.001	0.60
Gubner-Ungerleider criterion	13.9%	95.4%	<0.001	0.55

Table 4 shows multivariable logistic regression analysis of various ECG LVH criteria and echocardiographic LVH. The three ECG LVH criteria were associated with echocardiographic LVH. Cornell voltage had the strongest association (OR: 2.51, p < 0.001), followed by GU (OR: 1.91, p = 0.002), and the SKL criterion (OR: 1.87, p < 0.001).

**Table 4:** Multivariable logistic regression analysis of ECG LVH criteria and echocardiographic LVH. Overall model (p = 0.045), Hosmer and Lemeshow (p = 0.183), Cox & Snell R-square (0.161), and Nagelkerke R-square (0.214). LVH: left ventricular hypertrophy.

Outcome (echocardiographic LVH)		
Co-variates	Adjusted odds ratio (95% CI)	P-value
Cornell voltage criterion	2.51 (1.78-3.53)	<0.001
Gubner-Ungerleider criterion	1.91 (1.26-2.90)	0.002
Sokolow-Lyon criterion	1.87 (1.41-2.48)	<0.001

Table 5 displays multivariable logistic regression analysis of ECG abnormalities and echocardiographic LVH. Prolonged QTc, LV strain pattern, LAD, PRWP, PVC, PAC, AFib, and LAA were all significantly associated with echocardiographic LVH (p < 0.05).

**Table 5:** Multivariable logistic regression analysis of ECG abnormalities and echocardiographic LVH. AFib: atrial fibrillation; LAA: left atrial abnormality; LAD: left axis deviation; LBBB: left bundle branch block; LVH: left ventricular hypertrophy; PAC: premature atrial contractions; PRWP: poor R wave progression; PVC: premature ventricular contractions. Overall model (p = 0.043), Hosmer and Lemeshow (p = 0.611), Cox & Snell R-square (0.063), and Nagelkerke R-square (0.085).

Outcome (echocardiographic LVH)		
Co-variates	Adjusted odds ratio (95% CI)	P-value
LAD	1.56 (1.09-2.24)	0.016
Prolonged QTc	1.68 (1.06-2.66)	0.028
Abnormal Q	1.77 (0.69-4.53)	0.234
Strain pattern	1.83 (1.23-2.72)	0.003
PRWP	2.36 (1.40-3.97)	0.001
LBBB	5.96 (0.70-50.64)	0.102
PVC	1.80 (1.10-2.91)	0.018
PAC	2.06 (1.10-3.87)	0.024
AFib	2.40 (1.20-4.82)	0.014
LAA	8.43 (2.95-24.05)	<0.001

## Discussion

Our study shows that ECG LVH criteria, independently or collectively, are the most commonly associated ECG abnormalities with echocardiographic LVH in our hypertensive cohort. We employed three of the most widely used classical ECG LVH criteria in clinical practice [13]. ECG LVH was followed by prolonged QTc, LV strain pattern, LAD, PRWP, PVC, PAC, AFib, and LAA. These non-amplitude ECG abnormalities are indicative of electrophysiological and structural changes due to increased LVM [6]. The higher prevalence of individual ECG LVH criteria over non-amplitude ECG abnormalities suggests that ECG LVH criteria remain a valuable research tool in enhancing the diagnostic capability of the ECG in detecting anatomic LVH or assessing CV risk. We, therefore, suggest that combining ECG LVH criteria with non-amplitude ECG abnormalities in diagnostic and CV risk scores may offer greater diagnostic and prognostic potential than using either independently.

Our study’s ECG LVH criteria demonstrated low sensitivity (13.9-27.1%) and high specificity (87.2-95.4%). This aligns with literature evidence of low sensitivity and high specificity in general and hypertensive populations when using echocardiography, regardless of ECG criteria or ethnicity [13-20]. Available data also show that when cardiac magnetic resonance (CMR) and computed tomography (CT) are used, the sensitivity is also low at 25-61% and 4-43%, and specificity is high at 75-95% and 85-97%, respectively [19,20]. Similarly, studies among Nigerian hypertensives, albeit with small sample sizes, have shown variable low sensitivity and high specificity [16-18].

In this hypertensive cohort, the Cornell voltage criterion showed the highest area under the ROC curve (0.60), outperforming GU and SKL voltage criteria. Similarly, Ricciardi et al. demonstrated that the Cornell voltage criterion had superior diagnostic performance compared to RaVF amplitude and the Peguero-Lo Presti criterion [21]. Likewise, Park et al. found Cornell voltage to have better performance over the SKL voltage criterion in their study of 332 Koreans [22]. However, Dada et al. in a Nigerian study found that SKL voltage and SKL-Rappaport voltage criteria combine the best sensitivity and specificity under the ROC compared to Romhilt-Estes and Cornell voltage criteria [16]. These divergent results on the performance of various ECG amplitude criteria support Bacharova and colleagues’ view that using the ECG to find the best criterion for LVH is impractical [6].

Prolonged QTc, which is a QRS pattern abnormality, was significantly associated with echocardiographic LVH in our hypertensive cohort. In the Losartan Intervention for Endpoint Reduction study (LIFE) by Oikarinen et al., increased LVMI and LVH were associated with prolonged QT interval and increased QT dispersion in hypertensive patients [23]. Other studies have found a significant relationship between prolonged QTc and LVMI, LVH, complex ventricular tachyarrhythmias, and mortality in hypertensive patients [24-26]. The association of LVH and prolonged QTc may partly explain the heightened mortality risk of LVH.

ECG LV strain pattern is a marker of LVH and adverse outcomes in population and hospital-based studies [27,28]. It has been reported to predict anatomic LVH even when SKL voltage did not [28]. In the LIFE study by Okin et al., strain pattern was associated with increased LV mass, relative wall thickness, and higher occurrence of echocardiographic LVH [27]. Beyond the association with echocardiographic LVH, Okin et al. also demonstrated that strain pattern is a significant predictor of CV mortality, myocardial infarction, and stroke in hypertensive patients [27].

Other QRS pattern abnormalities found in our cohort were LAD and PRWP. Although not as extensively studied as prolonged QTc and LV strain pattern, they have also been found to be associated with LVH [6].

AFib, PVCs, and PACs were significantly associated with echocardiographic LVH in our study. These arrhythmias are universally agreed to be associated with anatomic LVH and result from alteration in cardiac electrical properties [6,29].

In our hypertensive cohort, LAA was significantly associated with echocardiographic LVH. This finding aligns with the Third National Health and Nutrition Survey analysis of 4,077 hypertensive participants, which showed that the prevalence of ECG LVH, ECG LAA, and their concurrent presence were 3.6%, 2.7%, and 0.34%, respectively [30]. After a median follow-up of 14 years, the highest mortality was observed in those with both ECG LAA and ECG LVH [30].

A major strength of this study is the large Nigerian population that was studied. To our knowledge, this is the first study to investigate ECG abnormalities in hypertensives with echocardiographic LVH. It is important to note the limitations of this study. This is a single-center and hospital-based study, and therefore the findings may not be generalizable to the whole of Nigeria. Additionally, we did not prospectively evaluate QRS patterns in hypertensive individuals without anatomic LVH because of our retrospective cohort design, making it difficult to establish a cause-and-effect relationship. Our study can therefore be considered exploratory in nature and further research is necessary to confirm our findings.

## Conclusions

The ECG abnormalities significantly associated with echocardiographic LVH in hypertensive Nigerians were ECG LVH amplitude criteria, prolonged QTc, LV strain pattern, PRWP, LAD, AFib, PVC, PAC, and LAA. These represent both amplitude and non-amplitude abnormalities from electrophysiological and structural changes due to increased LVM. The low sensitivity of our ECG amplitude criteria supports the call for a shift away from finding the best ECG LVH criterion for diagnosing anatomic LVH. If a higher prevalence of ECG LVH criteria is confirmed in prospective, multi-center hospital or community-wide studies, consideration may be given to using both amplitude and non-amplitude abnormalities to enhance the sensitivity of ECG for diagnosing LVH and or assessing cardiovascular risk in hypertensive patients.

## References

[1] Mancia G, Kreutz R, Brunström M (2023). 2023 ESH guidelines for the management of arterial hypertension the Task Force for the Management of Arterial Hypertension of the European Society of Hypertension: endorsed by the International Society of Hypertension (ISH) and the European Renal Association (ERA). J Hypertens.

[2] de Simone G, Izzo R, Chinali M (2010). Does information on systolic and diastolic function improve prediction of a cardiovascular event by left ventricular hypertrophy in arterial hypertension?. Hypertension.

[3] Sundström J, Lind L, Arnlöv J, Zethelius B, Andrén B, Lithell HO (2001). Echocardiographic and electrocardiographic diagnoses of left ventricular hypertrophy predict mortality independently of each other in a population of elderly men. Circulation.

[4] De Bacquer D, De Backer G, Kornitzer M, Blackburn H (1998). Prognostic value of ECG findings for total, cardiovascular disease, and coronary heart disease death in men and women. Heart.

[5] Ang D, Lang C (2008). The prognostic value of the ECG in hypertension: where are we now?. J Hum Hypertens.

[6] Bacharova L, Chevalier P, Gorenek B (2023). ISE/ISHNE expert consensus statement on ECG diagnosis of left ventricular hypertrophy: the change of the paradigm. The joint paper of the International Society of Electrocardiology and the International Society for Holter Monitoring and Noninvasive Electrocardiology. J Electrocardiol.

[7] Kligfield P, Gettes LS, Bailey JJ (2007). Recommendations for the standardization and interpretation of the electrocardiogram: part I: the electrocardiogram and its technology a scientific statement from the American Heart Association Electrocardiography and Arrhythmias Committee, Council on Clinical Cardiology; the American College of Cardiology Foundation; and the Heart Rhythm Society endorsed by the International Society for Computerized Electrocardiology. J Am Coll Cardiol.

[8] Lang RM, Badano LP, Mor-Avi V (2015). Recommendations for cardiac chamber quantification by echocardiography in adults: an update from the American Society of Echocardiography and the European Association of Cardiovascular Imaging. J Am Soc Echocardiogr.

[9] Hancock EW, Deal BJ, Mirvis DM (2009). AHA/ACCF/HRS recommendations for the standardization and interpretation of the electrocardiogram: part V: electrocardiogram changes associated with cardiac chamber hypertrophy: a scientific statement from the American Heart Association Electrocardiography and Arrhythmias Committee, Council on Clinical Cardiology; the American College of Cardiology Foundation; and the Heart Rhythm Society: endorsed by the International Society for Computerized Electrocardiology. Circulation.

[10] Rautaharju PM, Surawicz B, Gettes LS (2009). AHA/ACCF/HRS recommendations for the standardization and interpretation of the electrocardiogram: part IV: the ST segment, T and U waves, and the QT interval: a scientific statement from the American Heart Association Electrocardiography and Arrhythmias Committee, Council on Clinical Cardiology; the American College of Cardiology Foundation; and the Heart Rhythm Society. Endorsed by the International Society for Computerized Electrocardiology. J Am Coll Cardiol.

[11] Postema PG, De Jong JS, Van der Bilt IA, Wilde AA (2008). Accurate electrocardiographic assessment of the QT interval: teach the tangent. Heart Rhythm.

[12] Luo S, Michler K, Johnston P, Macfarlane PW (2004). A comparison of commonly used QT correction formulae: the effect of heart rate on the QTc of normal ECGs. J Electrocardiol.

[13] Bacharova L, Schocken D, Estes EH, Strauss D (2014). The role of ECG in the diagnosis of left ventricular hypertrophy. Curr Cardiol Rev.

[14] Bressman M, Mazori AY, Shulman E (2020). Determination of sensitivity and specificity of electrocardiography for left ventricular hypertrophy in a large, diverse patient population. Am J Med.

[15] Wang D, Xu JZ, Zhang W (2020). Performance of electrocardiographic criteria for echocardiographically diagnosed left ventricular hypertrophy in Chinese hypertensive patients. Am J Hypertens.

[16] Dada A, Adebiyi AA, Aje A, Oladapo OO, Falase AO (2005). Standard electrocardiographic criteria for left ventricular hypertrophy in Nigerian hypertensives. Ethn Dis.

[17] Dada A, Adebiyi AA, Aje A, Oladapo OO, Falase AO (2006). Comparison of Araoye's criteria with standard electrocardiographic criteria for diagnosis of left ventricular hypertrophy in Nigerian hypertensives. West Afr J Med.

[18] Ogunlade O, Akintomide AO (2013). Assessment of voltage criteria for left ventricular hypertrophy in adult hypertensives in south-western Nigeria. J Cardiovasc Dis Res.

[19] Krittayaphong R, Nomsawadi V, Muenkaew M, Miniphan M, Yindeengam A, Udompunturak S (2013). Accuracy of ECG criteria for the diagnosis of left ventricular hypertrophy: a comparison with magnetic resonance imaging. J Med Assoc Thai.

[20] Truong QA, Ptaszek LM, Charipar EM (2010). Performance of electrocardiographic criteria for left ventricular hypertrophy as compared with cardiac computed tomography: from the Rule Out Myocardial Infarction Using Computer Assisted Tomography trial. J Hypertens.

[21] Ricciardi D, Vetta G, Nenna A (2020). Current diagnostic ECG criteria for left ventricular hypertrophy: is it time to change paradigm in the analysis of data?. J Cardiovasc Med (Hagerstown).

[22] Park JK, Shin JH, Kim SH (2012). A comparison of Cornell and Sokolow-Lyon electrocardiographic criteria for left ventricular hypertrophy in Korean patients. Korean Circ J.

[23] Oikarinen L, Nieminen MS, Viitasalo M (2001). Relation of QT interval and QT dispersion to echocardiographic left ventricular hypertrophy and geometric pattern in hypertensive patients. The LIFE study. The Losartan Intervention For Endpoint Reduction. J Hypertens.

[24] Nadarajah R, Patel PA, Tayebjee MH (2021). Is hypertensive left ventricular hypertrophy a cause of sustained ventricular arrhythmias in humans?. J Hum Hypertens.

[25] Kulan K, Ural D, Komsuoğlu B, Ağaçdiken A, Göldeli O, Komsuoğlu SS (1998). Significance of QTc prolongation on ventricular arrhythmias in patients with left ventricular hypertrophy secondary to essential hypertension. Int J Cardiol.

[26] Haugaa KH, Bos JM, Borkenhagen EJ, Tarrell RF, Morlan BW, Caraballo PJ, Ackerman MJ (2014). Impact of left ventricular hypertrophy on QT prolongation and associated mortality. Heart Rhythm.

[27] Okin PM, Devereux RB, Nieminen MS (2004). Electrocardiographic strain pattern and prediction of cardiovascular morbidity and mortality in hypertensive patients. Hypertension.

[28] Ehara S, Hasegawa T, Matsumoto K (2014). The strain pattern, and not Sokolow-Lyon electrocardiographic voltage criteria, is independently associated with anatomic left ventricular hypertrophy. Heart Vessels.

[29] Bayés-Genís A, Guindo J, Viñolas X, Tomás L, Elosua R, Duran I, Bayés de Luna A (1995). Cardiac arrhythmias and left ventricular hypertrophy in systemic hypertension and their influences on prognosis. Am J Cardiol.

[30] Ahmad MI, Mujtaba M, Anees MA, Li Y, Soliman EZ (2019). Interrelation between electrocardiographic left atrial abnormality, left ventricular hypertrophy, and mortality in participants with hypertension. Am J Cardiol.

